# Insulin Signaling Regulates Mitochondrial Function in Pancreatic β-Cells

**DOI:** 10.1371/journal.pone.0007983

**Published:** 2009-11-24

**Authors:** Siming Liu, Terumasa Okada, Anke Assmann, Jamie Soto, Chong Wee Liew, Heiko Bugger, Orian S. Shirihai, E. Dale Abel, Rohit N. Kulkarni

**Affiliations:** 1 Department of Medicine, Harvard Medical School, Boston, Massachusetts, United States of America; 2 Division of Endocrinology, Metabolism and Diabetes and Program in Molecular Medicine, University of Utah School of Medicine, Salt Lake City, Utah, United States of America; 3 Department of Medicine, Boston University School of Medicine, Boston, Massachusetts, United States of America; 4 Research Division, Joslin Diabetes Center, Harvard Medical School, Boston, Massachusetts, United States of America; University of Bremen, Germany

## Abstract

Insulin/IGF-I signaling regulates the metabolism of most mammalian tissues including pancreatic islets. To dissect the mechanisms linking insulin signaling with mitochondrial function, we first identified a mitochondria-tethering complex in β-cells that included glucokinase (GK), and the pro-apoptotic protein, BAD_S_. Mitochondria isolated from β-cells derived from β-cell specific insulin receptor knockout (βIRKO) mice exhibited reduced BAD_S_, GK and protein kinase A in the complex, and attenuated function. Similar alterations were evident in islets from patients with type 2 diabetes. Decreased mitochondrial GK activity in βIRKOs could be explained, in part, by reduced expression and altered phosphorylation of BAD_S_. The elevated phosphorylation of p70S6K and JNK1 was likely due to compensatory increase in IGF-1 receptor expression. Re-expression of insulin receptors in βIRKO cells partially restored the stoichiometry of the complex and mitochondrial function. These data indicate that insulin signaling regulates mitochondrial function and have implications for β-cell dysfunction in type 2 diabetes.

## Introduction

Insulin and insulin like growth factor I (IGF-I) play critical roles in triggering pleiotropic actions including metabolism, proliferation and survival in most mammalian cells including the pancreatic islets [1]. The two hormones bind to their cognate receptors and activate Akt/protein kinase B via the phosphatidylinositol 3-kinase (PI3-K)-generated polyphospho-phosphatidylinositol pathway to phosphorylate the protein BAD in the regulation of apoptosis [2]. Indeed, we and several other laboratories have used cell line, primary islet and genetically engineered mouse models to demonstrate that insulin/IGF-I signaling plays a critical role in the regulation of hormone secretion, proliferation and survival of pancreatic β-cells (see review [3]). Among the various models, it is notable that mice with β-cell specific knockout of insulin receptors (βIRKO) and/or IGF-1 receptors demonstrate reduced expression of the glucokinase (GK) gene and blunted glucose-stimulated insulin secretion [4], [5], [6], [7]. βIRKO mice also display an age-dependent decrease in islet mass, poor compensatory islet response to high fat-diet induced islet growth and increased susceptibility to diabetes, suggesting a role for insulin signaling in the regulation of β-cell survival [8].

While the precise defect in β-cell growth and/or function that triggers overt diabetes is not fully understood, mitochondrial dysfunction in β-cells has been implicated as one factor that impairs β-cell survival and function [9]. Mitochondria play a major role in insulin secretion [10], [11] and in β-cell apoptosis [12], [13] triggered by diverse internal or external cues ultimately promoting cell death [14]. Indeed, islets from patients with type 2 diabetes exhibit low ATP content and a blunted glucose-stimulated insulin secretion (GSIS) and increased mitochondrial volume [9], together implicating a role for mitochondria in the pathogenesis of β-cell dysfunction.

Growth factor-mediated pathways have been reported to modulate mitochondrial function and regulate the activity of the pro-apoptotic protein, BAD [15]. Interestingly, studies in hepatocytes and β-cells have shown the presence of a BAD/glucokinase complex at the mitochondria suggesting a point of intersection for processes that regulate glycolysis and apoptosis [16]. Further, hyperglycemia has been reported to modulate the levels of GK and alter its interactions with mitochondria leading to apoptosis of β-cells [17], [18]. Considering the critical role of glucokinase in cellular metabolism and survival, and our previous observations of reduced expression of GK in β-cells lacking insulin or IGF-1 receptors [5], [19], we undertook the current study to explore whether absence of insulin signaling in β-cells impacts mitochondrial function.

In this study, we report the presence of a BAD/GK complex at the mitochondria in mouse and human β-cells. Furthermore, we provide evidence that lack of insulin receptors in β-cells impacts localization of BAD_S_, alters the stoichiometry of the BAD/GK complex and modulates phosphorylation of BAD_S_ that together contribute to defects in mitochondrial function.

## Results

### Identification of BAD/glucokinase complex in β-cell mitochondria

**Figure 1:**
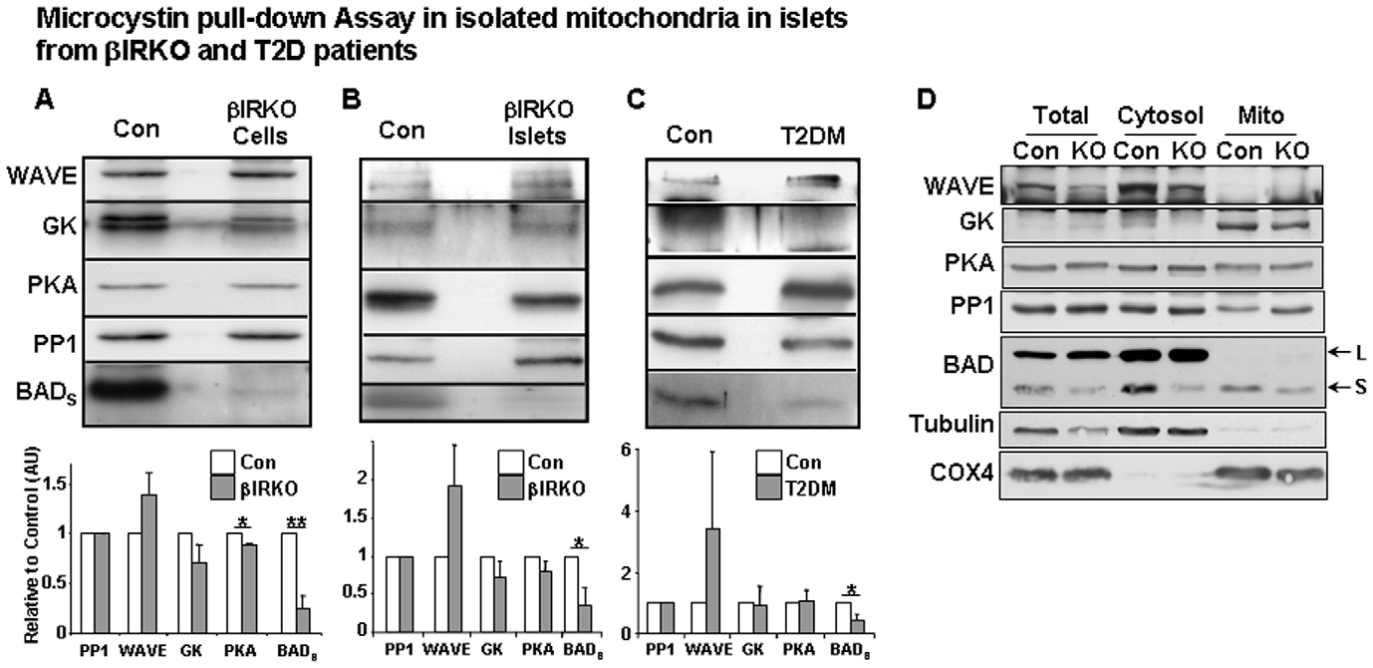
Presence of BAD/GK complex in mitochondria. Upper panels: Western blotting of five components in the BAD/GK complex with the indicated antibodies. Lower panels: Mean data after quantitation. Each component was normalized to PP1 and control samples were set to an arbitrary level of 1. A, control (Con) and βIRKO β-cell lines. B, control and βIRKO islet cells. C, islet cells from controls or patients with type 2 diabetes. Representative of 3–5 independent experiments. * p<0.05; ** p<0.001 versus Control. Open bar: controls; Shaded bar: βIRKO or type 2 diabetes β-cells. WAVE1, A protein kinase A scaffold; GK, glucokinase; PP1, protein phosphotase 1; PKA, protein kinase A; BAD_S_, BCL2-antagonist of cell death. D, Western blotting of five components in BAD/GK complex in cytosolic and mitochondrial compartments fractionated from control (Con) or βIRKO (KO) β-cell lines. Equal amount of proteins was loaded in each lane. Using total amount of protein loaded for the cytosol and mitochondria ∼20% of total cellular GK is associated with mitochondria. L and S denote the large and small isoforms of BAD respectively.

To explore the presence of a putative BAD/GK complex in β cells, we isolated mitochondria from β-cell lines, or islets derived from control or βIRKO mice and from diabetic and non-diabetic human subjects. Mitochondria were subjected to microcystin pull down assays. Similar to descriptions of the mitochondrial complex in hepatocytes [16], we detected five proteins: WAVE1 (a protein kinase A scaffold protein), glucokinase (GK), protein kinase A (PKA), protein phosphatase 1 (PP1), and the pro-apoptotic protein BAD_S_ (Fig. 1A–C). All components of the BAD/GK complex were identified in cultured β-cells and in mouse and human islets, indicating that this complex is conserved across species. Evaluation of the distribution of each component in cytosolic and mitochondrial fractions revealed that WAVE1 exists largely in the cytosol and mitochondrial WAVE1 comprises a small proportion of cellular WAVE protein (Fig. 1D). Interestingly, we observed robust expression of glucokinase in mitochondrial fractions when equal amounts of protein from mitochondria and cytososl were loaded (Fig. 1D). However, it is likely that the amount of GK that resides in the cytosol is greater than at the mitochondria (Fig. 1D). Among the BAD isoforms [17], BAD_L_ largely localized to the cytosol while BAD_S_ was identified in both cytosolic and mitochondrial fractions. On the other hand, PKA and PP1 were detectable in both cytosol and mitochondria (Fig. 1D).

Assessment of the effects of absence of insulin receptors or of insulin resistance on the complex, revealed a significant reduction in BAD_S_ in mitochondrial samples from β-cell lines or islets isolated from βIRKO mice and in islets isolated from humans with diabetes (75% of control, p<0.05) (Fig. 1A–C and Fig. S4). In mitochondria from β-cell lines isolated from βIRKO mice, PKA was reduced (12% of control, p<0.05) (Fig. 1A), and we also observed a trend towards a decrease in GK protein (29% of control, p = 0.08) in the mutants. Together, these data indicate that absence of insulin signaling in β-cells (βIRKO) or the diabetic state in humans is linked to reduced BAD_S_ in the mitochondrial complex.

### BAD_S_ phosphorylation, p70S6K and JNK signaling

BAD_S_, a pro-apoptotic protein belonging to the Bcl-2 family, can be phosphorylated in response to growth factors at three sites, Ser-112, Ser-136 or Ser-155 [20]. Kinases implicated in activating serine phosphorylation on BAD, include Akt/protein kinase B, p70S6K, PKA, Rsk, and PAK1 [20]. To investigate the role of insulin versus IGF-I in mediating BAD_S_ phosphorylation in β-cells, we stimulated β-cells with either hormone and examined mitochondrial or cytosolic fractions. For all experiments, the purity of the fractions was confirmed (Fig. S1).

#### βIRKO mitochondria exhibit decreased BAD_S_ but increased Ser-112 and Ser-136 BAD_S_

**Figure 2:**
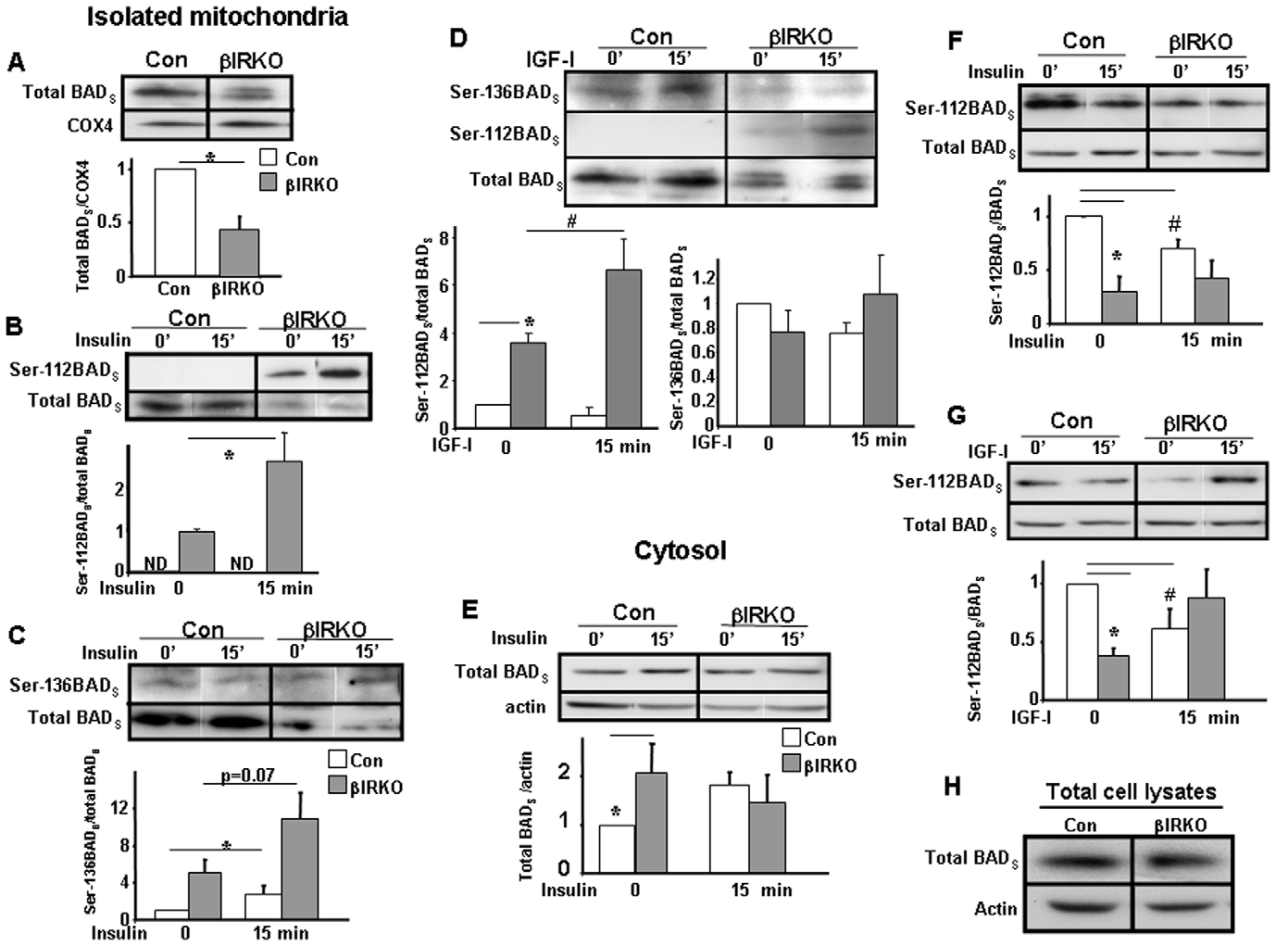
Altered expression of BAD_S_ and phospho-BAD_S_ in mitochondria and cytosol. Control or βIRKO cells were treated with 100 nM of either insulin or IGF-I for 15 min and fractionated mitochondria and cytosol were examined. Protein levels were assessed by densitometry. Controls were set to an arbitrary evel of 1. **In Figures A though G, the upper panels show representative Western blots and lower panels show mean values after quantitation of data. In H a representative Western blot is shown. Figures A, B, C and D relate to **alterations in isolated mitochondrial fractions and Figures E, F, and G relate to alterations in cytosolic fractions. A, Reduced total BAD_S_, * p<0.05 βIRKO vs control cells. B, Mitochondrial BADs phosphorylation at Ser-112, * p≤0.05, basal vs insulin stimulation, n = 3. ND, non-detectable. C, Mitochondrial BADs phosphorylation at Ser-136, * p≤0.05, basal vs insulin stimulation, n = 3. D, Mitochondrial BADs phosphorylation at Ser-112 and Ser-136 under basal or IGF-I stimulated conditions, * p≤0.05 vs. Control basal; # p<0.05, βIRKO**±**IGF-I; n = 3. Open bar: controls; Shaded bar: βIRKO. E, Total cytosolic BAD_S,_ * p<0.05. F and G, Ser-112 phosphorylation pattern of cytosolic BAD_S_ upon insulin (F) or IGF-I (G) treatments. * p<0.05 control basal vs βIRKO basal; # p<0.05, control±insulin/IGF-I. H, Total BAD in whole cell lysates. Open bar: controls; Shaded bar: βIRKO.

Consistent with reduced BAD_S_ protein in the BAD/GK complex, BAD_S_ content was reduced in mitochondria isolated from βIRKO cells (Fig. 2A). Phosphorylation of BAD_S_ at Ser-112 was increased in βIRKO mitochondria but was virtually undetectable in control cells. Furthermore insulin stimulation increased Ser-112 BAD_S_ in βIRKO mitochondria (Fig. 2B), which likely reflects insulin signaling via intact IGF-1 receptors ([21] and Fig. 2D).

Relative to total levels of BAD_S_, basal levels of Ser-136 phosphorylation of BAD_S_ were also increased in βIRKO mitochondria versus controls, and phosphorylation tended to increase further with insulin treatment (Fig. 2C). Compared to changes in Ser-112 phosphorylation, these changes were not as dramatic (Fig. S2A, B). Given the low abundance of BAD_S_ in the βIRKO mitochondria we conclude that the majority of the remaining BAD_S_ was in the phosphorylated state. The phosphorylation of BAD_S_ is unlikely to involve Akt since its activation is extremely low in βIRKO cells (see below). On the contrary, phosphorylation of BAD is likely due to enhanced IGF-I signaling in βIRKO cells (see below) that potentially activates RSK1, which in turn, has been reported to phosphorylate BAD_S_ at both Ser-112 and 136 [22]. In support of this hypothesis, and consistent with enhanced IGF-I signaling in βIRKO cells, we observed increased Ser-112 phosphorylation of BAD_S_ in βIRKO but not in control cells following incubation with IGF-I (Fig. 2D).

#### βIRKO cytosol expresses increased BAD_S_ and decreased Ser-112 BAD_S_

To evaluate relative changes in cytosol versus mitochondria, we next examined signaling events in cytosolic fractions. In βIRKO cytosol, the pattern of BAD_S_ expression and phosphorylation were different than that observed in mitochondria. Thus, total BAD_S_ showed a trend towards an increase but Ser-112 phosphorylation was reduced in basal conditions (Fig. 2E,F,G). The mitochondrial and cytosolic data collectively suggest that BAD_S_ is redistributed away from the mitochondria in the cytosol in βIRKO cells without a change in the total cellular content of BAD_S_. This was confirmed following analysis of whole cell lysates showing no differences in total BAD_S_ between βIRKO and control cells (Fig. 2H and Fig. S2A). Cytosolic BAD_S_ protein content tended to increase in insulin stimulated control cells, but did not change in βIRKO cells. One possible explanation for these observations is that increased Akt activation, in control relative to βIRKO cells results in increased phosphorylation of BAD_S_ at Ser-136 leading to sequestration of Ser-136 BAD_S_ with 14-3-3 proteins in the cytoplasm of controls (Fig. S2A) [23]. Therefore, relative to βIRKO cells, there is more cytosolic Ser-136 BADs in control cells, which is consistent with increased Akt-mediated phosphorylation and cytosolic sequestration in control cells.

In the cytosolic fractions extracted from control cells, we observed a significant decrease in Ser-112 phosphorylation of BAD_S_ in response to either insulin or IGF-I stimulation (Fig. 2F,G). The mechanism for this attenuation is uncertain, but these observations raise the possibility that Ser-112 phosphorylation might be negatively regulated by insulin or IGF-I mediated activation of Akt. βIRKO cytosol contains less Ser-112 BAD_S_ compared to control (Fig. 2F,G), which may suggest that phosphorylation of Ser-112 BAD_S_, may preferentially occur at the mitochondria (Fig. 2B and Fig. S2A). Indeed, Ser-112 phosphorylation of BAD_S_ in βIRKO cytosol did not increase following insulin treatment. In contrast, IGF-I treatment increased Ser-112 phosphorylation in βIRKO cells (Fig. 2D) (p<0.05), suggesting that the IGF-I signaling module that activates BAD, might be hyperactivated in βIRKO cells (Fig. 2F,G).

#### βIRKO cells show increased mitochondrial P70S6K and JNK1 activation

**Figure 3:**
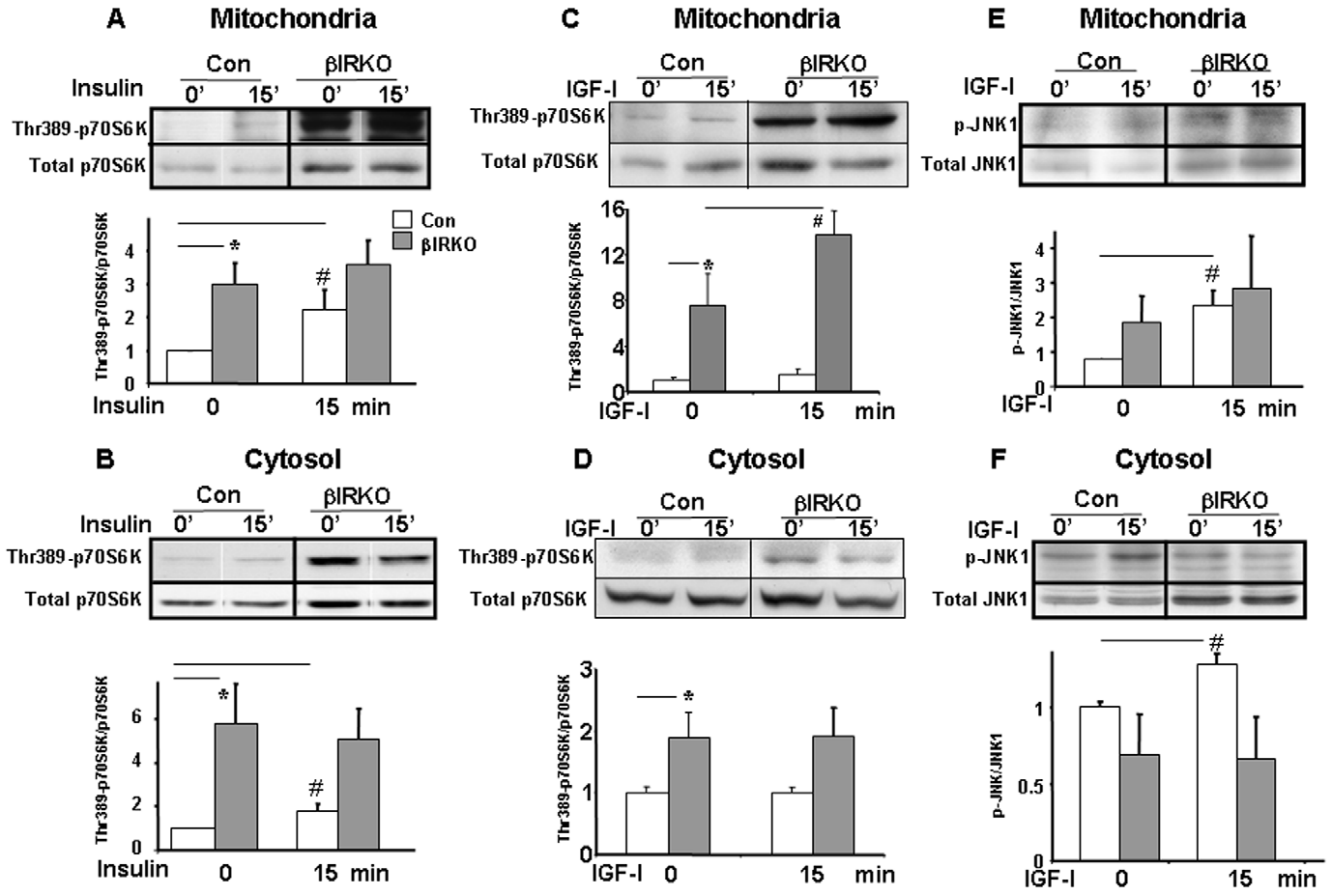
Altered phospho-p70S6K and phospho-JNK1 activation in mitochondria. Control or βIRKO cells were treated with 100 nM of either insulin or IGF-I for 15 min and fractionated cytosol and mitochondria were used for analyses. Controls were set to an arbitrary level of 1. In each Figure, the upper panel shows the representative Western blot and lower panel shows the mean value after quantitation of data. A, Responses in mitochondrial p70S6K to insulin treatment. * p<0.05; # p≤0.05. B, Responses in cytosolic p70S6K to insulin treatment. * p = 0.06; # p = 0.078; C and D, Responses in mitochondrial and cytosolic p70S6K to IGF-I treatment in control and βIRKO cells. * p<0.05; # p<0.05. E and F, Responses in mitochondrial and cytosolic JNK1 to IGF-I treatment in control and βIRKO cells. # p<0.05. Open bar: controls; Shaded bar: βIRKO.

We next explored potential mechanisms that could account for the preferential increase in phosphorylation of BAD in mitochondria of βIRKO mice. Consistent with the existence of mitochondria-tethered p70S6K in Rat-1a cells [24], we also detected this kinase in mitochondria in β-cells. Indeed, similar to other insulin sensitive cells, insulin stimulation of control cells increased phosphorylation of mitochondrial p70S6K (Fig. 3A).

βIRKO mitochondria exhibited higher basal activation of Thr-389 p70S6K in the mitochondrial and cytosolic fractions, which did not increase any further following insulin stimulation (Fig. 3A, B). It is possible that this reflects the consequence of increased basal IGF-I signaling, particularly at the level of the mitochondria, acting via Akt-independent signaling pathways such as MAPK [25], which we believe provides a potential explanation for the elevated phosphorylation of Ser-112 and Ser-136 of BAD_S_ in βIRKO mitochondria [24]. Indeed, in isolated mitochondria, addition of IGF-I significantly increased Thr-389 phosphorylation of p70S6K in the βIRKOs but had minimal effect in the controls (Fig. 3C). In contrast, IGF-I did not further increase cytosolic Thr-389 phosphorylation of p70S6K beyond the elevated basal levels in βIRKO (Fig. 3D).

The JNK (c-Jun N-terminal kinase) pathway is activated by diverse stressors and can influence multiple cellular processes. Because mitochondrial dysfunction could activate stress signaling in β-cells, we investigated JNK activity in mitochondria. JNK1 phosphorylation and total JNK1 protein was increased in mitochondria from βIRKO cells (Fig. 3E), which is consistent with the report that JNK co-localizes with mitochondria through its interacting protein [26]. The trend towards increased basal JNK1 phosphorylation levels in mitochondria, but not in cytosolic fractions, suggests that JNK1 activation might be linked to the stress induced by dysfunctional mitochondria in βIRKO cells (Fig. 3E,F). In control cells, IGF-I but not insulin, triggered phosphorylation of JNK1 in both mitochondrial and cytosolic fractions (Fig. 3E,F). Thus, the JNK/MAPK pathway is more responsive to IGF-I than to insulin in β-cells as has been suggested in other cell types [27]. The lack of a similar effect of IGF-I in βIRKO cells could be due to high basal activation of JNK1 that precludes further activation (Fig. 3E,F).

Given the role of the BAD/GK complex in the regulation of glycolysis and apoptosis [16], and our observations of changes in the BAD/GK complex in βIRKO mitochondria, we reasoned that these changes could be associated with impaired mitochondrial function in βIRKO cells.

### Altered mitochondrial function in βIRKO β-cells and islets

#### Blunted glucose-stimulated mitochondrial membrane potential response in βIRKO β-cells and islets

**Figure 4:**
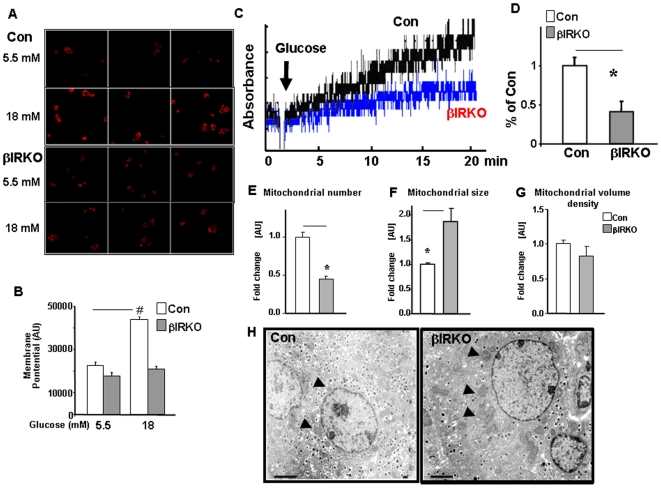
Altered mitochondrial function in βIRKO β-cells. A–B, Reduced mitochondrial membrane potential response to glucose stimulation in βIRKO β-cells. A, dispersed islet β-cells from control or βIRKO mice were stained by TMRE and analyzed by confocal microscopy. B, Fluorescence intensity in images was quantitated by NIH ImageJ. n = 3–5; # p<0.05, 5.5 mM vs 18 mM glucose. Open bar: controls; shaded bar: βIRKO β-cells. C–D, Reduced mitochondrial glucokinase activity in βIRKO β-cells. Glucokinase activity in freshly purified mitochondria from either control or βIRKO cells is plotted as absorbance of glucose-6-phosphate dehydrogenase-driven increase in NADPH fluorescence (C). The activity is illustrated by the rates determined by measuring optical density changes over linear periods, between 5 and 20 minutes (D). Mean of three independent experiments is shown. The activities of controls were set to an arbitrary level of 1. * p<0.05. E–G, Altered mitochondrial number and size. Quantitative analyses of mitochondrial ultrastructrue in islet β-cells from control or βIRKO mice. Mitochondrial number (E), size (F), and volume density (G) were quantitated as described in Methods, * p<0.05, n = 5. H, Representative transmission electron microscopy (TEM) micrographs of islet β-cells from control or βIRKO mice. Arrowheads point mitochondria showing increased size in βIRKOs. n = 28–30 cells from 3–5 mice. Magnification bar: 2 µM

Mitochondria respond to glucose stimulation in a dose-dependent manner by altering their membrane potential and mitochondrial metabolism, which plays a determinant role in glucose-stimulated insulin secretion [28], [29]. To assess the membrane potential response in islets *ex vivo*, we performed experiments in dispersed islet β-cells derived from control or βIRKO mice using the mitochondrial dye (TMRE). β-cells isolated from βIRKO islets exhibited significantly reduced membrane potential response to glucose stimulation compared to controls (Fig. 4A,B) and similar results were observed in βIRKO cell lines (Fig. S3A).

#### Decreased mitochondrial GK activity in βIRKO β-cells

Increased mitochondrial membrane potential results from increased glycolysis and oxidative phosphorylation. We hypothesized that altered mitochondrial glucokinase activity contributes to the poor membrane potential response to glucose stimulation. We therefore measured glucokinase activity in mitochondria using the G6PDH-coupled enzyme assay. Freshly purified mitochondria from control cells showed a time-dependent increase in NADPH generation and glucokinase enzyme activity in response to glucose stimulation while there was significantly less NADPH generation in mitochondria from βIRKO cells (Fig. 4C,D). NADPH generation was not observed following treatment with the non-metabolized analogue, 2-deoxy glucose, confirming specificity of the assay (data not shown). Thus, decreased level of BAD_S_ in the BAD/GK complex potentially leads to reduced mitochondrial GK activity in βIRKO cells, consistent with the reports in hepatocytes [16].

#### Decreased mitochondrial number and increased mitochondrial size in βIRKO β-cells

A potential cause of the decreased mitochondrial membrane potential response to glucose is altered mitochondrial mass [30]. Electron microscopy revealed that mitochondria in βIRKOs were rounded (Fig. 4H, arrows) and larger than those in control islet cells (Fig. 4F and H), but the number of mitochondria was reduced (Fig. 4E). The mitochondrial volume densities, however, were not different between the groups (Fig. 4G). Mitotracker staining of βIRKO cell lines revealed fluorescence consistent with no reduction in mitochondrial mass (Fig. S3B).

#### Increased mitochondrial oxygen consumption but decreased cellular ATP content in βIRKO β-cells

**Figure 5:**
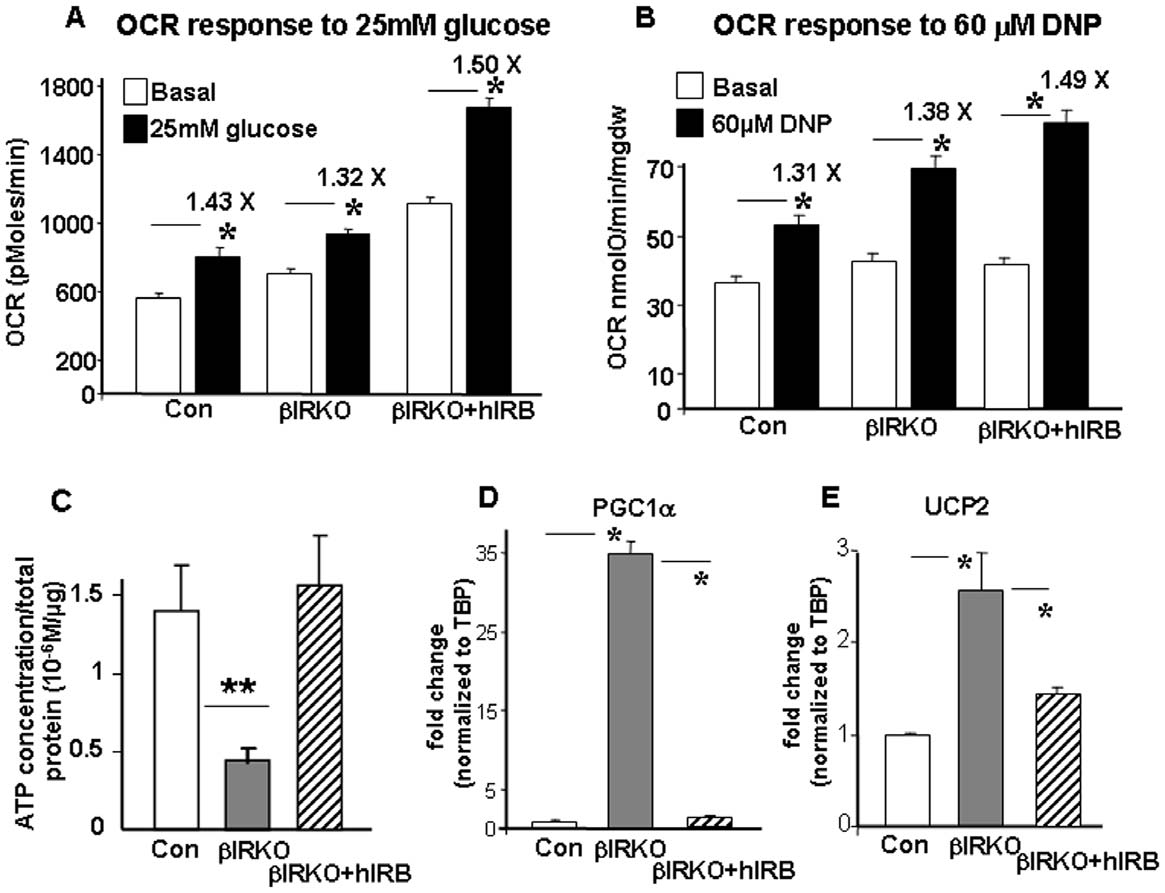
Re-expression of insulin receptors improved altered mitochondrial function in βIRKO β-cells. A, Basal and 25 mM glucose stimulated oxygen consumption rates (OCR) in control, βIRKO or βIRKO cells re-expressing insulin receptors (βIRKO+hIRB) cells, * p<0.05, n = 6. B, OCR response to 60 µM DNP (2,4-Dinitrophenol) in control, βIRKO or βIRKO+hIRB cells. OCRs were normalized by cell numbers in all assays, * p<0.05, n = 22. C, Re-expression of insulin receptors recovered the reduction of ATP production in βIRKO cells in response to 25 mM glucose stimulation. After starvation, control, βIRKO or βIRKO+hIRB cells were treated with 2 mM and 25 mM glucose in KRB buffer for 30 min, ATP was extracted and measured, ** p<0.001, n = 3. D and E, Re-expression of insulin receptors reduced the PGC1α and UCP2 expression to control levels. Expression levels of PGC1α (D) and UCP2 (E) were assessed in control, βIRKO or βIRKO+hIRB cells using real-time PCR. All cells grown in normal culture conditions; mRNA was extracted and cDNA was synthesized for real-time PCR, * p<0.05, n = 3.

To further explore mechanisms that contribute to the lower mitochondrial membrane potential in the βIRKOs, we measured oxygen consumption responses to treatment with glucose or DNP (2,4-dinitrophenol). Surprisingly, both control and βIRKO cells responded with a similar rise in oxygen consumption to either stimulus (Fig. 5A, B). Despite similar increases in oxygen consumption in glucose-stimulated control and βIRKO cells, there was a dramatic reduction in ATP content in glucose-stimulated βIRKO cells (Fig. 5C). The increase in oxygen consumption, accompanied by reduced ATP generation, and a blunted increase in membrane potential suggests that βIRKO mitochondria are significantly uncoupled. UCP2 expression was increased in βIRKO cells (Fig. 5E) and could represent one mechanism for mitochondrial uncoupling in these cells, similar to that observed in other models of type 2 diabetes [31]. Interestingly, expression levels of PGC-1α in the βIRKO cells were markedly increased, which likely represents a compensatory response to dysfunctional mitochondria (Fig. 5D). Recent studies have shown that PGC-1α and PGC-1β are transcriptional regulators of UCP2, and that increased PGC-1α/β - mediated upregulation of UCP2 may contribute to β cell dysfunction [32], [33].

### Rescue of phenotype by re-expression of insulin receptors in βIRKOs

**Figure 6:**
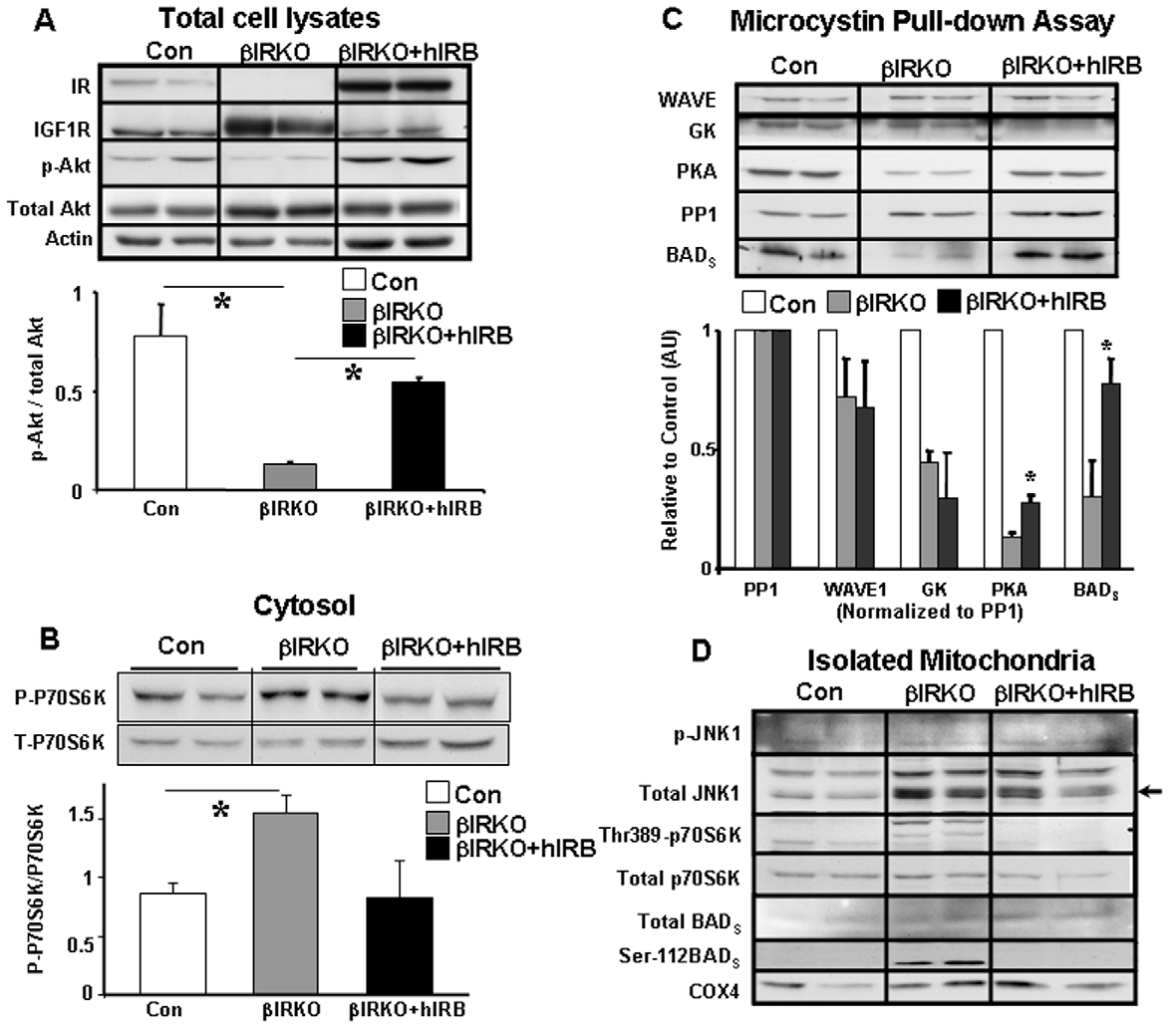
Normalization of signaling complexes after re-expression of insulin receptor B isoforms in βIRKO β-cells. A, Upper panel, Protein profile after expression of insulin receptors in βIRKO cells was immunoblotted by antibodies as indicated. p-Akt of controls were set to an arbitrary level of 1, * p<0.05, n = 3. Lower panel shows quantitation of p-Akt to total Akt. B, Upper panel, P70S6K and phosphor-P70S6K were assessed in cytosolic fractions from control, βIRKO or βIRKO cells re-expressing insulin receptors (βIRKO+hIRB), p-P70S6K of controls were set to an arbitrary level of 1, * p<0.05, n = 3. Lower panel shows quantitation of p-P70S6K to total p70S6K. C, Upper panel, microcystin pull down assays to demonstrate the five protein components of BAD/GK complex in mitochondria isolated from control, βIRKO or βIRKO cells re-expressing insulin receptors (βIRKO+hIRB), * p<0.05. Lower panel, each component was normalized to PP1 which was set to an arbitrary level of 1 for each cell type. D, Mitochondrial fractions isolated from control, βIRKO or βIRKO cells re-expressing insulin receptors (βIRKO+hIRB) were used to evaluate phosphorylation of JNK1, p70S6K, BAD_S_. Arrow indicates total JNK1.

To confirm that the defects in mitochondria in βIRKO β-cells are due to disrupted insulin signaling, we re-expressed the human insulin receptor-B isoform in βIRKO β-cells and revaluated the mitochondrial BAD/GK complex and mitochondrial function. In βIRKO cells re-expressing insulin receptors, Akt phosphorylation was restored to ∼70% of control levels (Fig. 6A) and the enhancedexpression of IGF-1 receptors and phospho-p70S6K were normalized to control levels (Fig. 6A,B). In microcystin pull-down assays, we observed a near-complete restoration of BAD_S_ in the BAD/GK complex, while PKA was significantly higher in the re-expressors compared to βIRKO fractions (Fig. 6C). Mitochondrial Thr-389 p70S6K and Ser-112 BAD phosphorylation were normalized in the absence of equivalent changes in phosphorylated JNK (Fig. 6D). However, the level of expression of GK in the complex was not completely restored suggesting that re-establishing insulin/Akt signaling only partially improves the stoichiometry of the BAD/GK complex in βIRKO β-cells.

In keeping with normalization of signaling complexes, re-expression of insulin receptors in βIRKOs increased oxygen consumption rates even further following stimulation with glucose or DNP (Fig. 5A,B). ATP levels and mitochondrial membrane potential showed an increase in glucose-stimulated cells (Fig. 5C and Fig. S3A) and expression levels of PGC-1α and UCP2 were restored to control levels (Fig. 5D,E). Thus re-expression of insulin receptors restored mitochondrial energetics in βIRKO cells in part by increasing mitochondrial coupling.

## Discussion

In this study we examined the significance of insulin signaling on mitochondrial function in pancreatic β-cells. We report that disruption of insulin signaling in β-cells alters the stoichiometry of a mitochondrial BAD/GK complex, reduces GK activity and promotes mitochondrial dysfunction. Re-expression of the insulin receptor in β-cells that lack insulin receptors largely restores the complex and improves mitochondrial function, highlighting a significant role for insulin signaling in the maintenance of the BAD/GK complex and in the regulation of mitochondrial function.

The identification of a BAD/GK complex in both mouse and human β-cells in this study indicates conservation across species and supports the concept of a potential functional link between apoptosis and glycolysis at the mitochondria, which is similar to observations in hepatocytes [16]. Indeed, the WAVE protein in the complex has been suggested to anchor GK and BAD to the mitochondria to efficiently co-ordinate spatial and temporal aspects of glucose metabolism [34]. The alterations in the expression of different components in the complex in mitochondria from β-cells from βIRKO provide potential insights into the altered function of β-cells and may explain some of the phenotypes observed in βIRKO mice [4], [6]. Similar to effects observed in BAD-deficient hepatocytes, decreased BAD_S_ protein in the βIRKO cells likely contributes to mitochondrial dysfunction and β-cell dysfunction, which may contribute to the glucose intolerance in both of these mutants [16], [35]. The BAD/GK complex was also disrupted in islets from humans with type 2 diabetes and showed an interesting similarity with islets from bIRKO mice. Thus it is possible that reduced BAD_S_ in the mitochondria may be linked to the blunted glucose response and altered mitochondrial ultra structure observed in islets isolated from patients with type 2 diabetes [9].

Our study provides evidence that altered insulin signaling is associated with mitochondrial function in the β-cell, as has recently been reported in cardiomyocytes [36]. The mechanism for mitochondrial dysfunction in βIRKO cells is multifactorial and includes reduced glucose-mediated augmentation in mitochondrial membrane potential and reduced ATP levels that could be due either to decreased synthesis or to increased consumption in order to maintain the membrane potential. Of interest, mitochondrial dysfunction occurs despite increased oxygen consumption. These observations suggest that despite reduced glucokinase activity and potentially reduced glucose delivery to βIRKO mitochondria they are also uncoupled. One potential mechanism for mitochondrial uncoupling is increased UCP2 expression, which may be driven by increased expression of PGC-1α and β [32], [33]. Moreover, increased UCP2 expression has been implicated in limiting stimulus-secretion coupling in β-cells in some animal models of diabetes but not in others [31], [37]. However, it is possible that this increased UCP2 expression may not contribute directly to mitochondrial dysfunction in βIRKO cells and it could be the result of ROS overproduction in response to glucose stimulation since it was found that deletion of insulin receptors in heart increases their propensity to enhance ROS when exposed to fatty acid substrates [36]. Also, DNP experiments suggest that the capacity of the OXPHOS machinery may not be intrinsically impaired in βIRKO cells; the improvement in oxygen consumption that we see in βIRKO and the rescued cells might be due to altered mitochondrial mass (Fig. S3B); that might increase total mitochondrial respiration. Together, the mitochondrial defects in βIRKO cells could be results of combined effects from reduced GK activity, ROS production and altered UCP2 expression. Finally, we also measured mitochondrial membrane potential response to glucose stimulation, and βIRKO cells that re-expressed the insulin receptors showed a trend towards improved membrane potential response in response to glucose (Fig. S3A), which is also consistent with the increased respiration. Although our experiments have not definitively identified all mechanisms that could account for mitochondrial dysfunction, our study clearly shows that impaired insulin signaling in β-cells fundamentally alters mitochondrial bioenergetics and function, which may ultimately impair insulin secretion.

Consistent with the growth factor-induced phosphorylation and translocation of BAD_S_ [20] we observed significant alterations in mitochondria isolated from β-cells lacking the insulin receptor. The high level of basal phosphorylation of Ser112-BAD and Ser136-BAD in the βIRKOs likely reflects the effects of signaling via the IGF-1 receptors. It is possible that the relative levels of activation of insulin versus IGF-1 receptors determine the overall phosphorylation state of BAD at the mitochondria, with Ser112 phosphorylation being specifically mediated by Akt-independent pathways, and Ser136 phosphorylation being activated by both Akt and non-Akt pathways [38].

Intact IGF-I signaling in βIRKO cells could mediate the activation of RSK and MSK1 to phosphorylate BAD at Ser-112 [22]. The phosphorylation state of BAD_S_ has also been reported to impact its binding with other proteins such as Bcl-XL [39]. Further, the silencing of all serine sites in the BAD^3SA/3SA^ mouse has been reported to reduce GK activity in hepatocytes while disruption of Ser-155 down-regulates GK activity in islets [16], [35]. These studies suggest that phosphorylation of BAD_S_ can modulate GK activity and impact apoptosis, likely by disruption of protein interactions in the complex. Our data suggest that the level of BAD_S_ phosphorylation correlates with function. This is reflected by the finding that βIRKO cells exhibit extremely high Ser112 phosphorylation in mitochondria (Fig. 2B and Fig. S2B) and phosphorylation can potentially interrupt the interaction between BAD_S_ and other proteins, including GK. Furthermore, re-expression of insulin receptors reversed the increase in Ser112 phosphorylation of BAD_S_ and increased the content of total BAD_S_ in the complex. Together, these data suggest the potential relevance of interactions between Ser112 phosphorylation and function of the complex. Based on the report by Danial et al [16], restoration of BAD_S_ in the complex would augment glucokinase function and mitochondrial metabolism. Additional studies such as mutagenesis of Ser112 of BAD_S_ will be necessary to prove a direct role of this phosphorylation event. In βIRKO mitochondria it is possible that phosphorylation at Ser-112 disrupts the residence of BAD_S_ in the mitochondrial complex and alters glucokinase activity and mitochondrial metabolism without affecting Ser-155-BAD (data not shown). These observations are consistent with the altered glucose sensing and altered apoptosis of β-cells in βIRKO mice [4], [5], [6]. The ability to reverse basal Ser-112 phoshorylation and restore BAD_S_ content to control levels by re-expressing the insulin receptor in βIRKO cells further underscores the role of insulin signaling in regulating the mitochondrial complex.

Another signaling pathway that could potentially influence the stoichiometry of the BAD/GK complex is the elevated expression of PTEN secondary to enhanced IGF-I signaling in βIKRO cells [40], [41]. Increased PIP2 could decrease the binding of PKA in the VCA domain of the WAVE protein, which is known to anchor GK and BAD to the mitochondria to efficiently coordinate spatial and temporal aspects of glucose metabolism [34], [42] and may contribute to the partial normalization of PKA content upon re-expressing the insulin receptor in βIRKO cells.

**Figure 7:**
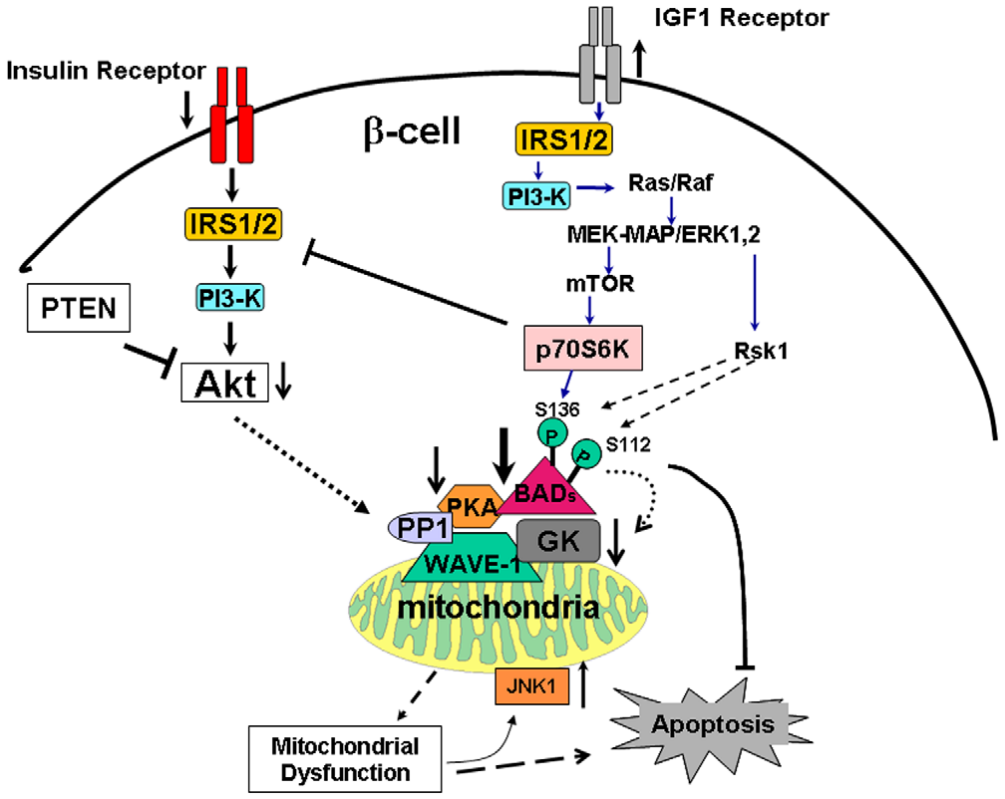
Schematic showing the impact of insulin/IGF-I signaling on β-cell mitochondrial metabolism and function. Impairment of insulin signaling alters BAD/GK complex and reduces mitochondrial function by inhibiting mitochondrial GK activity. The compensatory increase in IGF-I signaling can impact phosphorylation of mitochondrial BAD to influence glucokinase activity and the mitochondrial threshold to cell death cues; it can also impact mitochondrial p70S6K to phosphorylate BADs. Mitochondrial dysfunction due to impaired insulin signaling can activate mitochondrial JNK1, which can phosphorylate mitochondrial substrate, e.g. BADS, to influence mitochondrial function and sensitivity to stress-induced cell death.

In the cytosol, the elevated p70S6K activation in βIRKO cells could potentially serine-phosphorylate IRS1 and further dampen insulin-stimulation of Akt in βIRKO cells [43] (Fig. 7), and potentiate IGF-I mediated pathways in the mutant cells. Indeed, activation of Akt/PKB has been reported to inhibit IGF-I activation of MAPK and mTOR pathways via Raf1 kinase, while adenoviral knockdown of Akt/PKB in INS-1 cells leads to enhanced ERK1/ERK2 signaling that is independent of IGF-I signaling [25].

We observed high levels of JNK1 activation in mitochondria but not in cytosolic fractions in βIRKO β-cells, which is consistent with the report that JNK1 activation is linked to dysfunctional mitochondria [44]. The activation of JNK1 by IGF-I, but not by insulin, in both cytosolic and mitochondrial fractions in control β-cells, implicates a role for IGF-I in the stress signaling pathway and is consistent with the potentiation of cytokine-mediated JNK following long-term IGF-I treatment [45]. We suggest that JNK1 could potentially contribute to Ser-112 phosphorylation on BAD_S_ to affect mitochondrial metabolism and function (Fig. 7). Our observations are consistent with the notion that down-regulation of Akt does not influence basal mitochondrial respiration [46]. It will be useful to dissect further, how insulin and IGF-I signaling pathways differentially modulate MAPK and mTOR target proteins, such as p70S6K and JNK1, to regulate mitochondrial function in β-cells. Further work is also necessary to explore whether insulin/IGF-I signaling directly modulates PGC-1α to affect mitochondrial biogenesis and function [47], [48].

In summary, this study provides novel insights into the role of insulin signaling in the regulation of the BAD/GK complex, glycolytic enzyme activity and mitochondrial metabolism in pancreatic β-cells. Ser112-BAD_S_ and its upstream kinases may be potential targets for the maintenance of the BAD/GK complex that is necessary for normal mitochondrial function and the regulation of β-cell survival.

## Materials and Methods

### Animals and cell lines

Experiments were performed using the β-cell specific insulin receptor knockout mice (βIRKO) and Lox/Lox littermates as controls [6]. All mice were maintained at the Taconic Facility (NY) or Brandeis University (Waltham, MA). All experiments were performed in compliance with Joslin Diabetes Center and Brandeis University Animal Care and Use Committee regulations. The control and βIRKO β-cell lines were derived and maintained as described previously [49].

### Mouse and human pancreatic islets

Mouse islets were isolated and purified by collagenase digestion as described previously [6]. Isolated islets were cultured in RPMI media with 10% FBS and 5.5 mM glucose for 48 h before experiments. Islets from controls and patients with type 2 diabetes were obtained from ICR (Islet Cell Resources, CA) and cultured in Miami medium #1 (Mediatech, NJ) [50].

### Mitochondrial isolation

Mitochondria were isolated using conventional differential centrifugation [16]. Briefly, cell lines or islets were homogenized in mitochondrial lysis buffer (220 mM mannitol, 70 mM glucose, 10 mM HEPES, 1 mM EGTA, pH 7.4). Subsequently, the lysate was centrifuged at 700 g to separate nuclear and cytosolic fractions, the cytosolic fraction was further centrifuged at 7000 g at 4°C to pellet mitochondria. The isolated mitochondria were subjected either to microcystin capture assay (see below), Western blotting or mitochondrial glucokinase activity assay. For mitochondrial glucokinase activity assays, the isolated mitochondria were further purified through a 30%–70% percoll gradient by centrifugation at 20,000 rpm in a Beckman SW41 rotor for 40 min. The mitochondrial layer in the gradient was removed and washed twice and immediately used for activity assays.

### Microcystin capture assay

Isolated mitochondria from each respective β-cell line or from islets were solubilized in the lysis buffer containing 6 mM CHAPS, 150 mM NaCl, 0.01 M sodium phosphate (pH 7.2) and 2 mM EDTA. Based on the specific high affinity of microcystin for protein phosphatase 1 (PP1) (one of the components in the BAD/GK complex), the solubilized mitochondria were incubated with microcystin beads (Upstate, NY) overnight at 4°C to pull down the BAD/GK complex. After incubation, the microcystin beads were washed and boiled in protein sample buffer. The protein bound to the microcystin beads were resolved by SDS-PAGE gels and detected by Western blotting to identify the components precipitated on the beads.

### Western blotting

To evaluate the role of insulin or IGF-I signaling, cell lines were incubated with 100 nM of either insulin or IGF-I for 15 min at 37°C. The 15 min time point was chosen based on pilot experiments to induce signaling effects in mitochondria. Proteins were extracted from cells or islets as previously described [51]. The protein concentration of each sample was determined using a BCA kit (Pierce Biotechnology, Holmdel, NJ). The extracted proteins were resolved by SDS-PAGE, and the protein was transferred to PVDF membrane (Millipore, MA). Western blotting results were normalized to actin and quantitated by densitometry. Antibodies to the following proteins were used: Phospho-BAD, BAD, phospho-p70S6K, p70S6K, phospho-JNK1, JNK1 (Cell Signaling Technology, Boston, MA), glucokinase and protein phosphotase 1 (PP1) (Santa Cruz Biotechnology, Santa Cruz, CA), PKA (Pharmingen, San Jose, CA) and evaluated in cytosol and mitochondrial fractions.

### Glucose-stimulated mitochondrial membrane potential response in β-cell lines and dispersed islet β-cells

β-cells were dispersed from islets of control or βIRKO mice as previously described [52]. Dispersed islet cells were cultured in RMPI medium with 10% FBS, antibiotics, 5.5 mM glucose on microscope coverslips and allowed to recover for 48 h. The dispersed islet cells were stained with insulin and glucagon antibodies to confirm purity of β-cells. Cells were stained with 10 nM TMRE (Molecular probe, Carlsbad, CA) for 45 min prior to glucose stimulation. Fluorescence intensity of cells was initially recorded in the basal condition followed by stimulation with 18 mM glucose. The altered fluorescence of treated cells was recorded under a confocal microscope (Zeiss, Germany) within 10 min. The fluorescence intensity in basal and treated conditions was quantitated by NIH Image J software (n = 60 to 80 cells). The mitochondrial membrane potential change was calculated by averaging fluorescence intensities before and after stimulation.

### Mitochondrial glucokinase activity

Mitochondrial glucokinase activity was measured using a glucose-6-phosphate dehydrogenase (G6PDH)-coupled enzyme assay [53]. The total NADPH generated reflects glucokinase activity in mitochondria. Briefly, ∼3 mg of isolated mitochondria was resuspended in reaction buffer containing 50 mM triethanolamine hydrochloride, 20 mM MgCl_2_, 100 mM KCl, 1 mM dithiothreitol, 0.1% bovine serum albumin, 10 mM ATP, 1 mM NADP and 4 µg/ml glucose-6-phosphate dehydrogenase (Sigma, St. Louis, MO). Then, 90 mM glucose was added to initiate the reaction and monitored by absorbance at 340 nm for 20 min at RT. In order to decrease NADPH background generated from mitochondrial respiration, all reactions were performed in the presence of mitochondrial respiration inhibitors (4 µM Rotenone, 100 nM Antimycin A and 2 µg/ml Oligomycin).

### Electron microscopy and determination of mitochondrial number, size and volume density

Islets were fixed with 2.5% glutaraldehyde at RT for 2 h. Ultra-thin sections (∼60 nm) were cut and stained with uranyl acetate and lead citrate before being examined under JOEL Transmission Electron Microscope (Tokyo, Japan). At least five islets were chosen randomly from control or βIRKO mouse islets and ∼4 randomly selected β-cells from each islet were photographed. At least 15 islet β-cells were examined from control or βIRKO islets for number and morphology of the mitochondria. Mitochondrial volume density and number were analyzed by stereology in a blinded fashion using the point counting method, as described previously [54]. Five representative images were analyzed per genotype, and four grids per image were analyzed. Average size of the mitochondria was calculated by dividing mitochondrial volume density by mitochondrial number.

### Re-expression of insulin receptor in βIRKO cells

Retroviral-packaging ∅ (Phoenix cells carrying the retroviral vector was kindly provided by C. R. Kahn, MD (Joslin Diabetes Center, Boston, MA). The vector encoding human insulin receptor B was obtained from I. Leibiger, Ph.D (Karolinska Institute, Stockholm, Sweden). Briefly, empty vector or vector expressing the insulin receptor was transfected using lipofectamine (Invitrogen, Carlsbad, CA) in Ø cells. The day after transfection, DMEM medium, supplemented with 10% fetal bovine serum (FBS) and 50**µg/ml gentamicin (Gibco, Invitrogen, Carlsbad, CA) was replaced. Supernatant was collected 48 h post-transfection and subjected to ultracentrifugation using an SW-28 rotor at 20,000 rpm. The transparent pellet was resuspended in 500**µl phosphate buffered saline (PBS, pH 7.4), aliquoted and stored at −80°C until further use. βIRKO cells were transfected with retrovirus particles. Twenty four h after transfection, 400 µg/ml hygromycin (Gibco, Invitrogen, Carlsbad, CA) was added to select the cells in which the expression vector was successfully integrated.

### Measurement of Mitochondrial Respiration

Oxygen consumption rates (OCR) were measured in whole cells by using a fiber-optic oxygen sensing probe as previously described [55] or the Seahorse XF24. Briefly, cells were suspended at a concentration of 2×10^6^ cells/ml by a magnetic stirrer at 37°C in a volume of 1 ml of buffer TD (137 mM NaCl, 5 mM KCl, 0.7 mM Na2HPO4, 25 mM Tris-Cl, pH 7.4) and transferred to the oxygen chamber. Endogenous (basal) respiration was measured for 1 min. Glucose (final concentration 25 mM) or the uncoupler 2,4-dinitrophenol (DNP) was injected into the chamber (83 mM final), and stimulated rates of oxygen consumption were determined. The Seahorse machine (Seahorse Bioscience, Billerica, MA) was used to measure oxygen consumption rate in adherent cells *in situ* without trypsinization.

### ATP assay

Cells were seeded in 12-well plates and incubated for 36–48 h before incubation for an additional 16 h with 2 mM glucose in DMEM with 0.1% BSA, followed by 2 mM glucose in KRB buffer for one h. The cells were then either incubated in KRB buffer containing 2 mM glucose (basal concentration) or KRB buffer containing 25 mM glucose (stimulatory concentration) for 30 min. ATP was then extracted with 0.5% TCA solution for 15 min at 4°C. An aliquot of the TCA extracted samples were diluted 20X with 0.1 M Tris-HCl, pH 7.75 buffer just before ATP measurement with the ENLITEN® ATP Assay System Bioluminescence Detection Kit (Promega, Madison, WI) according to the manufacturer's recommendation. Total protein concentration was measured using the BCA protein Assay (Pierce, Rockford, IL), and ATP generated was normalized to total protein.

### Statistics

Data were analyzed by Student's ‘t’ test or analyses of variance (ANOVA) with post-hoc comparisons as appropriate. Unless otherwise indicated all experiments were performed on at least three independent occasions.

## Supporting Information

Figure S1 Mitochondrial purity assay. Mitochondria were isolated from β-cells as described in Materials and Methods. To examine the purity of either the mitochondrial or cytosolic fraction, a mitochondrial marker COX4 or a cytosolic marker actin were immunoblotted for each fraction.
(0.20 MB PPT)

Figure S2 Distribution of BAD and phospho-BAD_S_ in β cells. Control or βIRKO cells were treated with 100 nM insulin for 15 min and fractionated, cytosol, mitochondria and total lysates were examined. A, Ser136-BAD_S_, Ser112-BAD_S_ and total BAD_S_ were immunoblotted. Tubulin and COX4 were used as loading control for cytosol and mitochondria respectively. B, Magnified image for Ser112-BAD_S_ in cytosol, mitochondria and total cell lysate. L and S denote the isoforms of BAD_S_.
(0.15 MB PPT)

Figure S3 Altered mitochondrial membrane potential and mass. A. Control, βIRKO or βIRKO+hIRB (βIRKO cells re-expressing insulin receptors) cells were treated with 16.7 mM glucose and stained with TMRE. The mitochondrial membrane potential change was calculated by averaging fluorescence intensities before and after stimulation. *p<0.05, control vs. βIRKO; p = 0.068, βIRKO vs. βIRKO+hIRB; n = 4. B. Control, βIRKO or βIRKO cells re-expressing insulin receptors (βIRKO+hIRB) cells were stained by Mitotracker dye and analyzed by flow cytometry; *p<0.05, control vs. βIRKO, and βIRKO vs. βIRKO+hIRB; n = 3.
(0.07 MB PPT)

Figure S4 Western immunoblotting of the five components in BAD/GK complex in islets from patients with type 2 diabetes. Islets from three controls and three patients with type 2 diabetes were fractionated and mitochondrial fractions were assessed using a mycrocystin pull-down assay. The pull-down was used to detect the five components that constitute the BAD/GK complex, using antibodies indicated in the figure and described in Methods.
(0.43 MB PPT)
