# Supplementary figures and images for: Insulin Signaling Regulates Mitochondrial Function in Pancreatic β-Cells
Source: PLoS One. 2009 Nov 24;4(11):e7983. doi: 10.1371/journal.pone.0007983 (PMC2776992; doi:10.1371/journal.pone.0007983)

## Slide 1
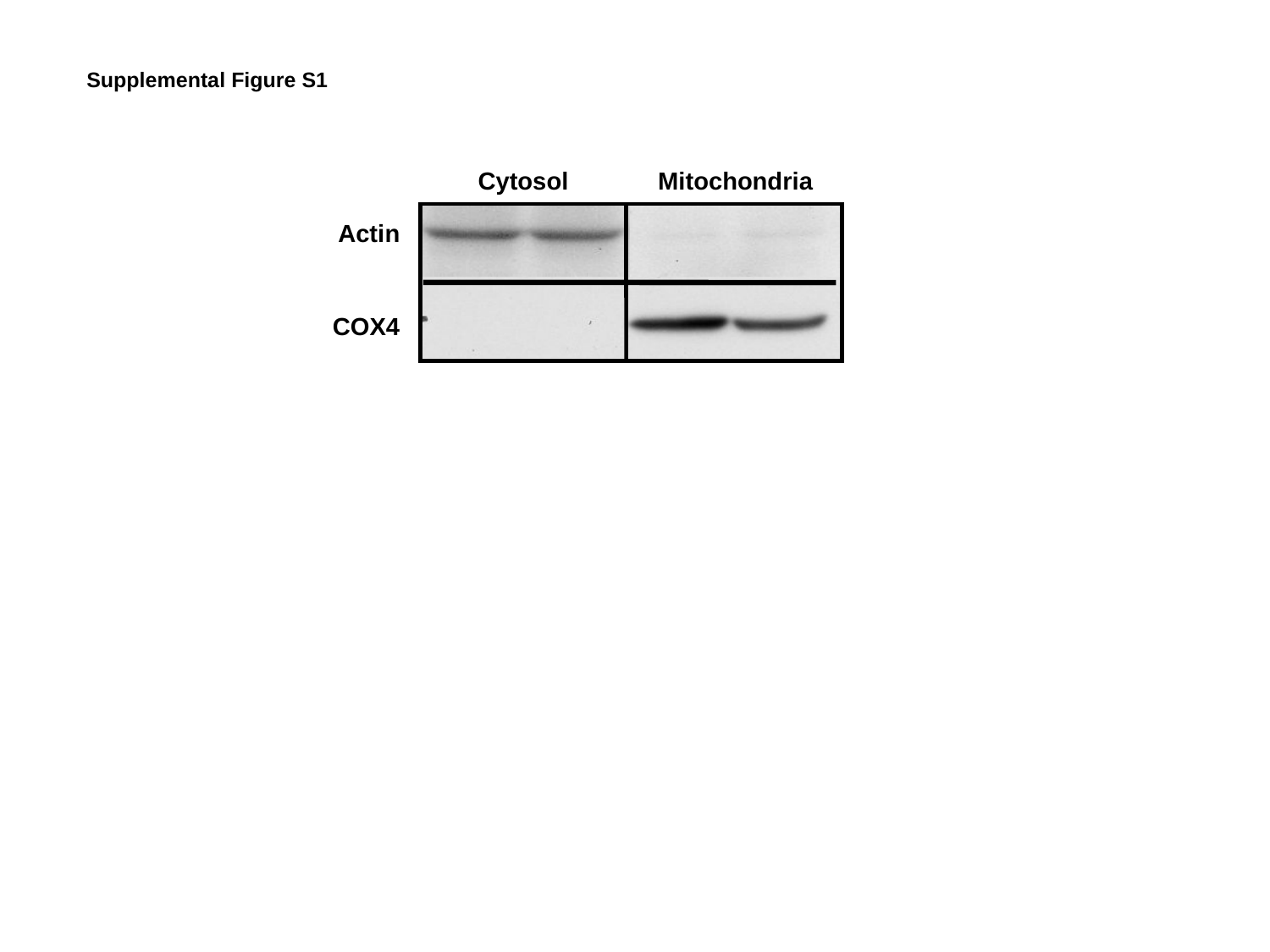

Supplemental Figure S1
Cytosol
Mitochondria
Actin
COX4

Supplement: Figure S1 — Mitochondrial purity assay. Mitochondria were isolated from β-cells as described in Materials and Methods. To examine the purity of either the mitochondrial or cytosolic fraction, a mitochondrial marker COX4 or a cytosolic marker actin were immunoblotted for each fraction. (0.20 MB PPT) [file pone.0007983.s001.ppt]
