# Supplementary material for: Insulin Signaling Regulates Mitochondrial Function in Pancreatic β-Cells
Source: PLoS One. 2009 Nov 24;4(11):e7983. doi: 10.1371/journal.pone.0007983 (PMC2776992; doi:10.1371/journal.pone.0007983)
Supplement: Figure S2 — Distribution of BAD and phospho-BADS in β cells. Control or βIRKO cells were treated with 100 nM insulin for 15 min and fractionated, cytosol, mitochondria and total lysates were examined. A, Ser136-BADS, Ser112-BADS and total BADS were immunoblotted. Tubulin and COX4 were used as loading control for cytosol and mitochondria respectively. B, Magnified image for Ser112-BADS in cytosol, mitochondria and total cell lysate. L and S denote the isoforms of BADS. (0.15 MB PPT) [file pone.0007983.s002.ppt]

## Slide 1
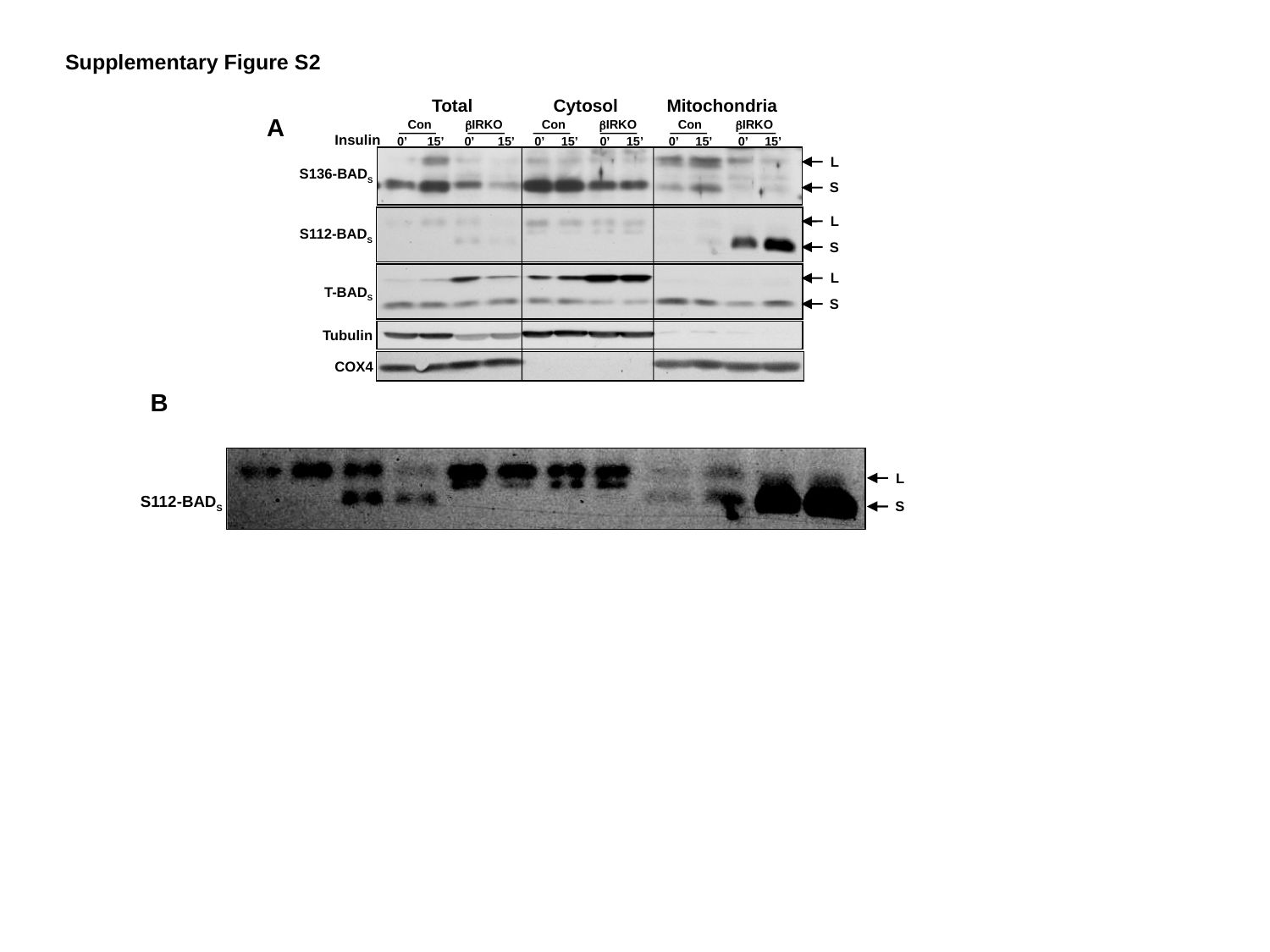

Supplementary Figure S2
Total
Cytosol
Mitochondria
A
Con βIRKO
Con βIRKO
Con βIRKO
Insulin
0’ 15’
0’ 15’
0’ 15’
0’ 15’
0’ 15’
0’ 15’
L
S136-BADS
S
L
S112-BADS
S
L
T-BADS
S
Tubulin
COX4
B
L
S112-BADS
S
