# Supplementary material for: Insulin Signaling Regulates Mitochondrial Function in Pancreatic β-Cells
Source: PLoS One. 2009 Nov 24;4(11):e7983. doi: 10.1371/journal.pone.0007983 (PMC2776992; doi:10.1371/journal.pone.0007983)
Supplement: Figure S3 — Altered mitochondrial membrane potential and mass. A. Control, βIRKO or βIRKO+hIRB (βIRKO cells re-expressing insulin receptors) cells were treated with 16.7 mM glucose and stained with TMRE. The mitochondrial membrane potential change was calculated by averaging fluorescence intensities before and after stimulation. *p<0.05, control vs. βIRKO; p = 0.068, βIRKO vs. βIRKO+hIRB; n = 4. B. Control, βIRKO or βIRKO cells re-expressing insulin receptors (βIRKO+hIRB) cells were stained by Mitotracker dye and analyzed by flow cytometry; *p<0.05, control vs. βIRKO, and βIRKO vs. βIRKO+hIRB; n = 3. (0.07 MB PPT) [file pone.0007983.s003.ppt]

## Slide 1
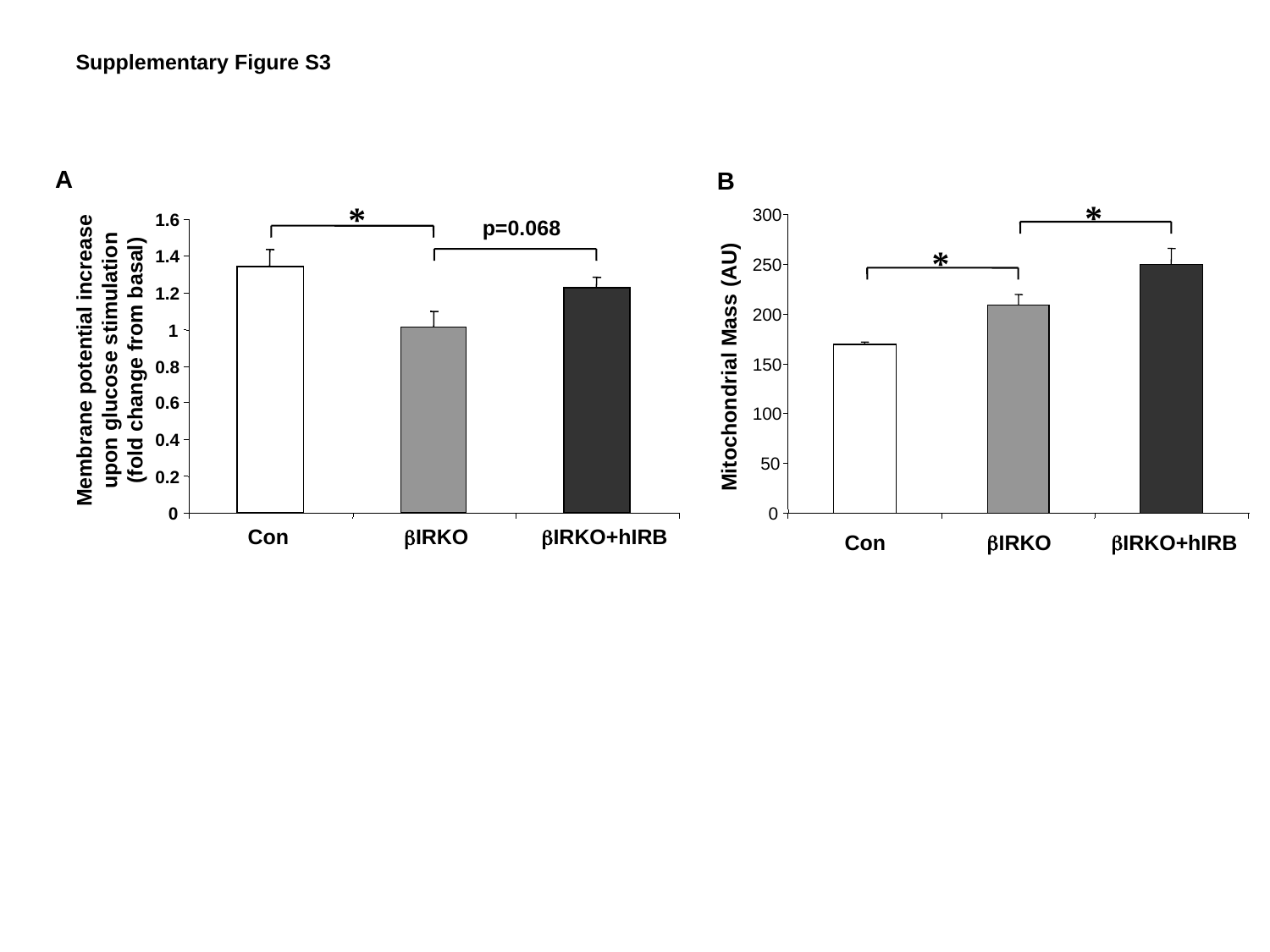

Supplementary Figure S3
A
B
*
*
300
p=0.068
1.6
*
1.4
250
1.2
200
1
Membrane potential increase upon glucose stimulation
(fold change from basal)
150
Mitochondrial Mass (AU)
0.8
0.6
100
0.4
50
0.2
0
0
Con
βIRKO
βIRKO+hIRB
Con
βIRKO
βIRKO+hIRB
