# Supplementary material for: Insulin Signaling Regulates Mitochondrial Function in Pancreatic β-Cells
Source: PLoS One. 2009 Nov 24;4(11):e7983. doi: 10.1371/journal.pone.0007983 (PMC2776992; doi:10.1371/journal.pone.0007983)
Supplement: Figure S4 — Western immunoblotting of the five components in BAD/GK complex in islets from patients with type 2 diabetes. Islets from three controls and three patients with type 2 diabetes were fractionated and mitochondrial fractions were assessed using a mycrocystin pull-down assay. The pull-down was used to detect the five components that constitute the BAD/GK complex, using antibodies indicated in the figure and described in Methods. (0.43 MB PPT) [file pone.0007983.s004.ppt]

## Slide 1
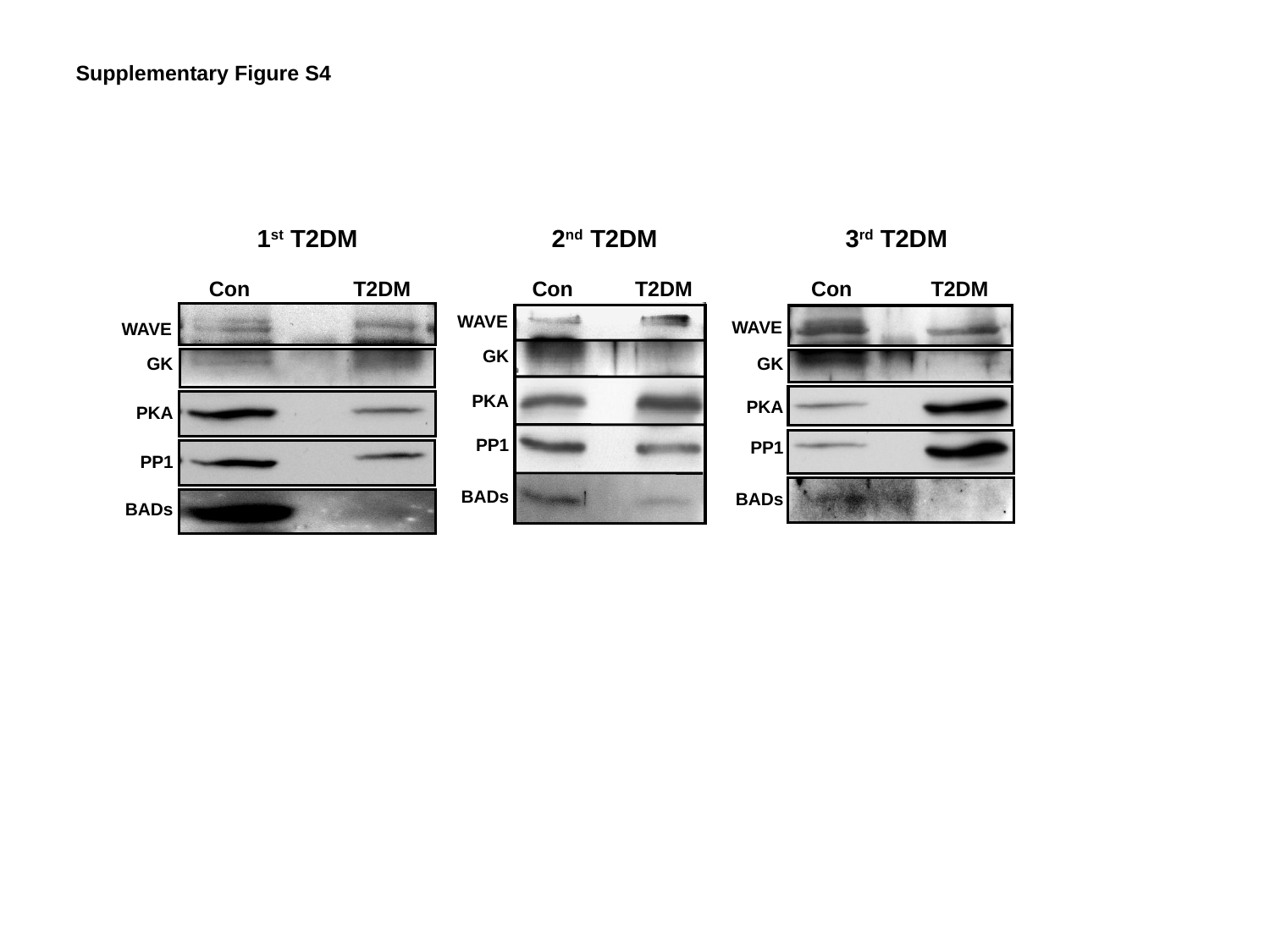

Supplementary Figure S4
1st T2DM
2nd T2DM
3rd T2DM
Con
T2DM
Con
T2DM
Con
T2DM
WAVE
WAVE
WAVE
GK
GK
GK
PKA
PKA
PKA
PP1
PP1
PP1
BADs
BADs
BADs
